# Fabrication and characterization of a novel catalyst based on modified zirconium metal-organic-framework for synthesis of polyhydroquinolines

**DOI:** 10.1038/s41598-023-43869-2

**Published:** 2023-10-03

**Authors:** Fatemeh Moghadaskhou, Reza Eivazzadeh-Keihan, Zahra Sadat, Azadeh Tadjarodi, Ali Maleki

**Affiliations:** 1 Research Laboratory of Inorganic Materials Synthesis, Department of Chemistry, Iran University of Science and Technology, Tehran, 16846-13114 Iran; 2 Catalysts and Organic Synthesis Research Laboratory, Department of Chemistry, Iran University of Science and Technology, Tehran, 16846-13114 Iran

**Keywords:** Chemistry, Materials science, Nanoscience and technology

## Abstract

A novel catalyst was fabricated in this study based on zirconium MOF modified with pyridine carboxaldehyde in a solvothermal reaction, embedded with cerium. In order to confirm the catalyst structure, various characterization techniques, including FTIR, Far IR, EDX, XRD, TGA, FE-SEM, ICP, and BET analyses, were employed. The results indicated that the UiO-66-Pyca-Ce (III) catalyst had a Langmuir surface area of 501.63 m^2^/g, a pore volume of 0.28 cm^3^/g, and a pore size of 2.27 nm. To study catalytic activity, a sequential approach of Knoevenagel condensation and Michael addition was used to synthesize various polyhydroquinoline derivatives. The reaction took place at ambient temperature. The UiO-66-Pyca-Ce (III) catalyst demonstrated high efficacy (90%) and reusability in asymmetric synthesis of polyhydroquinoline derivatives for several reasons, including the possession of three Lewis acid activation functions.

## Introduction

**Figure 1:**
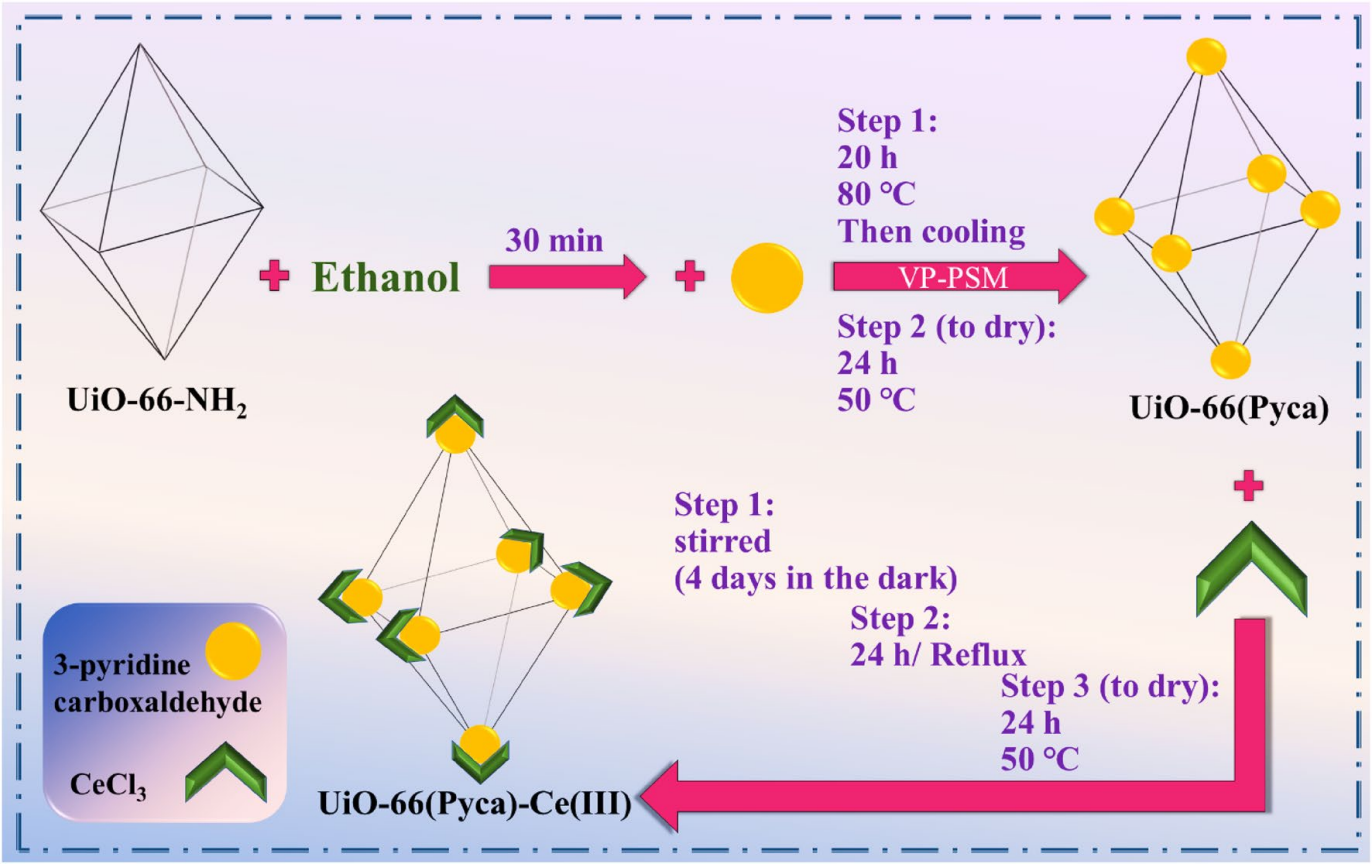
Preparation of MOF UiO-66-Pyca-Ce (III) correction reaction in 2 steps.

Recently, multi-component reactions have been employed to develop new methods of Synthesis strategies with abundant molecular diversity^1,2^. These strategies have resulted high atom economy and simple methods for synthesizing heterocyclic compounds^3^ which leading to biological active compounds like antituberculosis, anticancer, antipain, and antidiabetic agents. As a result, a wide variety of studies have been carried out on the popularization of the synthesis of these heterocyclic compounds using both heterogeneous and homogeneous catalysts^4–7^. In addition, the development of synthetic methods to fabricate N-heterocyclic polyhydroquinoline has been highlighted by researchers^8^. To prepare bioactive molecules, different types of catalysts, such as LiBr^9^, CTAB^10^, CuBr^11^, GO nanoparticles^12^, l-proline^13^, CTACl^14^ and salicylic acid^15^, have been utilized. Many of these reactions suffer from disadvantages like harsh reaction conditions, low product yields, and slow and tedious procedures, which all considered negative points. To solve these problems, scientists have developed and offered novel synthetic techniques. Recently; metal–organic frameworks (MOFs) that include metal-exo clusters and organic linkers have received more attention^16–18^. These MOFs are utilized in various applications because of their high porosity, highly effective surface, and chemical stability. These applications include drug delivery^19,20^, food storage^21^, gas absorption or storage^22^, hazardous gas storage^23^, chemical sensing^24^ and optoelectronics^25^. MOFs either have catalytic activity or used as a suitable support for catalysts which make them a good choice in catalysis applications^26–32^. Post-synthetic modification (PSM) method is used to functionalize MOFs which can improve their catalytic activity^33^. PSM can be performed without causing any negative effects on the framework’s stability. these modifications are resulted by adding new active sites in MOFs structure^34^ that enhance catalytic activities of these compounds. Another enhancement leads to easy separation and recovery of heterogeneous catalysts which highlighted MOFs as a reusable catalysts^35–44^. Cerium (III) chloride heptahydrate (CeCl_3_·7H_2_O), known as a promoter in organic synthesis, has attracted much attention due to its various applications^45^. Cerium halides have advantages such as being water resistance, user friendly, non-toxicity, and affordability. Furthermore, they can be reused multiple times without any purification^46^. They are recognized as effective Lewis acid catalysts^47^. In current study, catalytic activity of zirconium MOF from the UiO-66 family was investigated. Pyridine carbaldehyde was used to modify UiO-66-NH2 using a post-synthesis approach. The cerium was subsequently incorporated into UiO-66-Pyca. The asymmetric Hantzsch reaction was employed to evaluate the catalytic activity of UiO-66-Pyca-Ce (III). The catalyst showed recyclability and multiple reusability, which is very favorable for both the environment and the economic standpoint. The sufficiency and catalytic performance of this new UiO-66-Pyca-Ce (III) catalyst were assessed in the asymmetric Hantzsch condensation reaction for the synthesis of polyhydroquinoline derivatives (5a-f) (Fig. 1). It should be noted that the response conducted as part of this research is environmentally safe and no toxic waste is produced.

## Experimental

### Materials and physical techniques

All reagents for synthesis and analysis were commercially available from Aldrich and Merck Companies and were used without further purification. The infrared (IR) spectra were recorded using a Thermo Nicolet IR 100 FT-IR spectrometer. A field emission scanning electron microscope (FESEM), specifically the German-made ZEISS SIGMA VP model with a gold coating, was used to analyze the samples. Utilizing monochromatic Co-Kα (1.78897 Å) radiation and a Philips X’pert diffractometer, measurements of X-ray powder diffraction (XRD) were made. The N_2_ desorption/adsorption isotherms of the synthesized samples were obtained using the BET technique with a Microtrac Bel Corp Belsorp mini II instrument. The N_2_ adsorption isotherm at 77 K was measured using a Micromeritics ASAP 2030 surface area analyzer. Thermogravimetry (TGA) was performed to determine the thermal stability using an SDTQ600 V20.9 analyzer with a heating rate of 10 °C/min under airflow. The metal cerium ion loading of the catalyst was determined by inductively coupled plasma (ICP) analysis using a Vavian 715-ES instrument. Proton nuclear magnetic resonance (^1^H NMR) spectroscopy was conducted on a Varian UnityPlus 400 instrument.

### Synthesis of UiO-66-Pyca

The UiO-66-NH_2_ crystals were prepared according to the literature procedure^48–50^. The prepared UiO-66-NH_2_ (0.3 g) was dispersed in 30 mL of ethanol in an orbicular-bottomed flask and stirred for 30 min. Thereafter, 0.9 mL of pyridine carboxaldehyde was added to the mixture, and the sealed flask was transferred to an oil bath. The solution was stirred at 80 °C for 20 h. The precipitate was separated by centrifugation after the yellow solution was cooled to room temperature. The acquired products were washed with abundant ethanol. Finally, the obtained UiO-66-Pyca was dried in a vacuum oven at 50 °C for 24 h.

### Synthesis of UiO-66-Pyca-Ce (III)

UiO-66-Pyca (10 mg) was dispersed into 20 mL of ethanol, and the suspension was sonicated for 10 min. Then, 40 mg of CeCl_3_ was added, and the mixture was stirred at room temperature for four days in darkness. The mixture was refluxed at 80 °C for 24 h. The yellow precipitated material was separated using centrifugation, and it was washed with ethanol and acetone. Finally, the material was dried at 50 °C for 24 h. This compound was named UiO-66-Pyca-Ce (III).

### Catalyst reaction

#### General method for one-pot synthesis of polyhydroquinoline derivatives (5a-f), to assess the catalytic activity of the UiO-66-Pyca-Ce (III)

To investigate the catalytic activity of UiO-66-Pyca-Ce (III) in the one-pot synthesis of polyhydroquinoline derivatives under ambient temperature conditions, several substituted aldehydes (1 mmol), dimedone (1 mmol), ammonium acetate (2 mmol), and ethyl acetoacetate (1 mmol) were stirred inside a flask in the presence of UiO-66-Pyca-Ce (III) (0.001 g) as a catalyst and ethanol as a green solvent. A 5:1 n-hexane/ethyl acetate ratio was used to monitor the reaction’s progress using the thin layer chromatography (TLC). Once the reaction was complete, the catalyst was separated through filtration and then washing. Additionally, to purify the received product, each individual product was recrystallized in ethanol.

## Results and discussion

The Zr_6_O_4_(OH)_4_(BDC-NH_2_)_6_ clusters that make up the UiO-66-NH_2_ metal–organic framework which resulting a 3D structure, has special properties like high surface area (1187 m^2^/g^–1^), good stability in wide variety of solvents, acidic and basic aqueous media (pH values ranging from 1 to 9), and thermal stability^51^. Through post-synthetic modification (PSM), it is possible to create functionalized MOFs with multiple active sites^34,52^. The high activity and good recyclability of these heterogeneous catalysts have shown that MOFs are an appropriate catalytic support. In this study, zirconium (IV) chloride, 2-amino-1,4-benzene dicarboxylic acid, and N, N-dimethylformamide (DMF) were used in a solvothermal reaction to create UiO-66-NH_2_. Three Lewis acid activating functions as a catalyst’s special quality, help to promote the asymmetric synthesis of polyhydroquinoline derivatives at ambient temperature. The structural confirmation of UiO-66-Pyca-Ce (III) were interpreted using various types of analysis. The functional groups present in the compound structure were identified using the FTIR spectrum. The morphology and elemental composition were determined using FE-SEM images and EDX analysis, respectively. The crystalline phase of the material was analyzed using XRD, and its thermal stability was assessed through TGA analysis. In order to examine the surface and porosity of UiO-66-Pyca-Ce (III), BET analysis was also carried out. Additionally, ICP analysis was performed to identify the elements present in the composition and determine their concentration.

### Analysis

#### XRD patterns of UiO-66-NH_2_, UiO-66-Pyca, and UiO-66-Pyca-Ce (III)

One benefit of crystalline materials is that the crystallinity and structural integrity can be assessed using XRD after post-synthesis modification. Figure 3a–c shows the XRD patterns of UiO-66-NH2, UiO-66-Pyca, and UiO-66-Pyca-Ce as they were synthesized using the solvothermal method. The UiO-66-NH2 metal–organic framework's XRD pattern is consistent with those mentioned in references^49,53^. The compound's high crystallinity is also indicated by the presence of sharp peaks. The XRD patterns of UiO-66- Pyca and UiO-66-Pyca-Ce (Fig. 3b and c) show that the post-synthesis modification process did not alter the UiO-66- NH2 structure.

#### FTIR analysis of UiO-66-NH_2_, UiO-66-Pyca, and UiO-66-Pyca-Ce (III)

**Figure 2:**
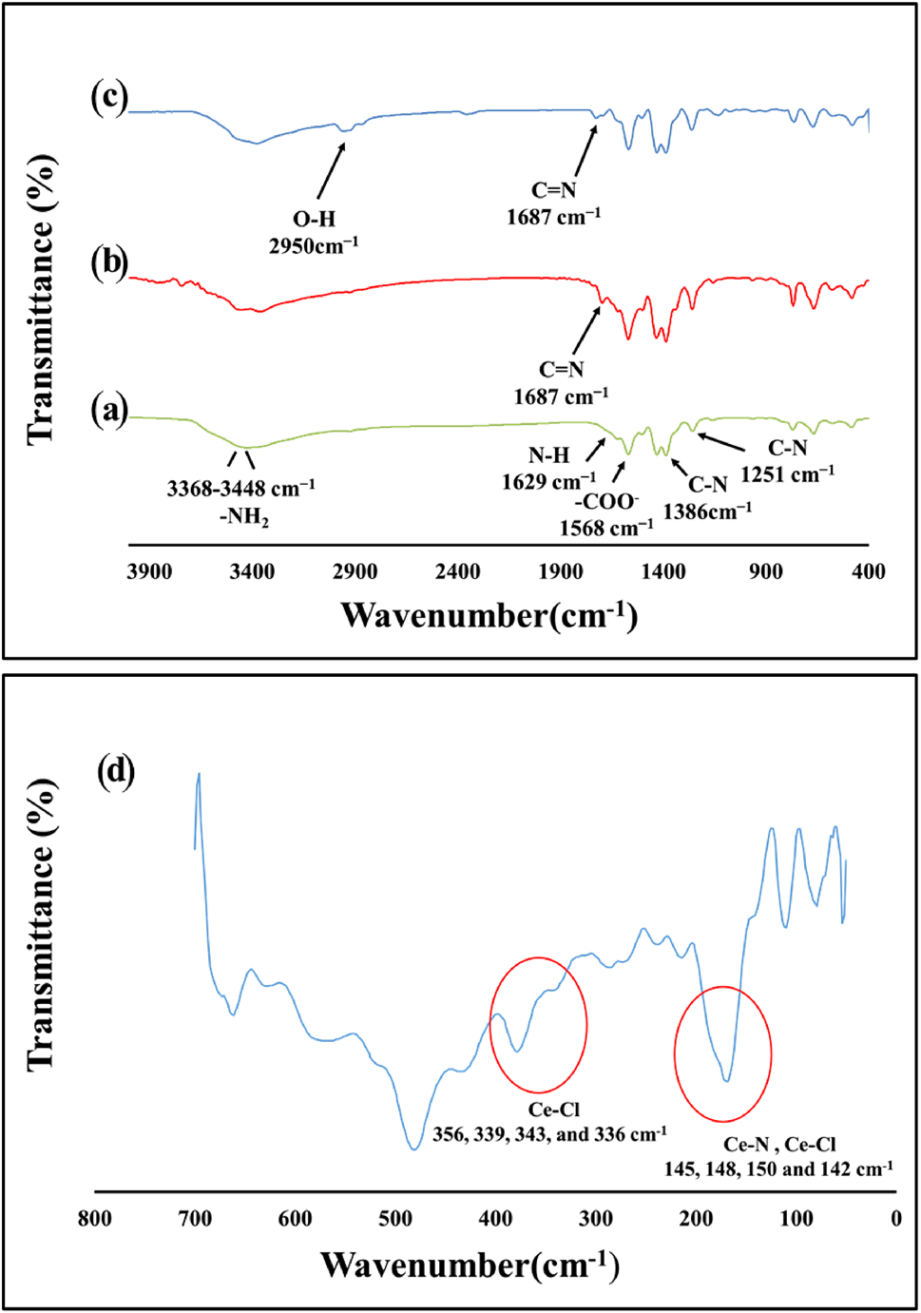
FTIR spectra of (a) the as-synthesized UiO-66-NH_2_, (b) UiO-66-Pyca, (c) UiO-66-Pyca-Ce (III) and (d) The Far IR spectrum of UiO-66-Pyca-Ce (III).

Figure 2 displays the FTIR spectra of the synthetic UiO-66-NH_2_, UiO-66-Pyca, and UiO-66-Pyca-Ce (III) compounds. The bands associated with the bending vibrations of N–H, the asymmetric and symmetric stretching vibrations of carboxylate (COO–), and the C–N stretching vibration of aromatic amines are found at 1568, 1629, 1386, and 1256 cm^–1^, respectively, in the FTIR spectrum of UiO-66-NH_2_ (Fig. 2a), which is consistent with the knowledge gleaned from prior studies^49^. A new band is seen at 1687 cm^–1^ in the FTIR spectra of UiO-66-Pyca and UiO-66-Pyca-Ce (III) (Fig. 2b and c), which can be attributed to the stretching vibration of the C=N bond caused by imine condensation of amino groups with the carbonyl group of pyridinecarbaldehyde, indicating the successful modification of the structure^49^. In the FTIR spectrum of UiO-66-Pyca-Ce (III), the bands associated with the asymmetric and symmetric stretching vibrations of N-Ce are observed at 360 and 460 cm^–1^, respectively, which are in agreement with the reported values in the literature^54^. To observe the peaks related to cerium, the spectrum in the range below 400 cm^–1^ should be checked. The closest vibration, VCe-Cl, appears at (356, 339, 343, and 336 cm^–1^) (m), while the tensile vibration VCe-N and the flexural vibration due to Cl are in the same band at (145, 148, 150, and 142 cm^–1^) (Fig. 2d)^54^.

#### ICP and TGA of UiO-66-Pyca-Ce (III)

**Figure 3:**
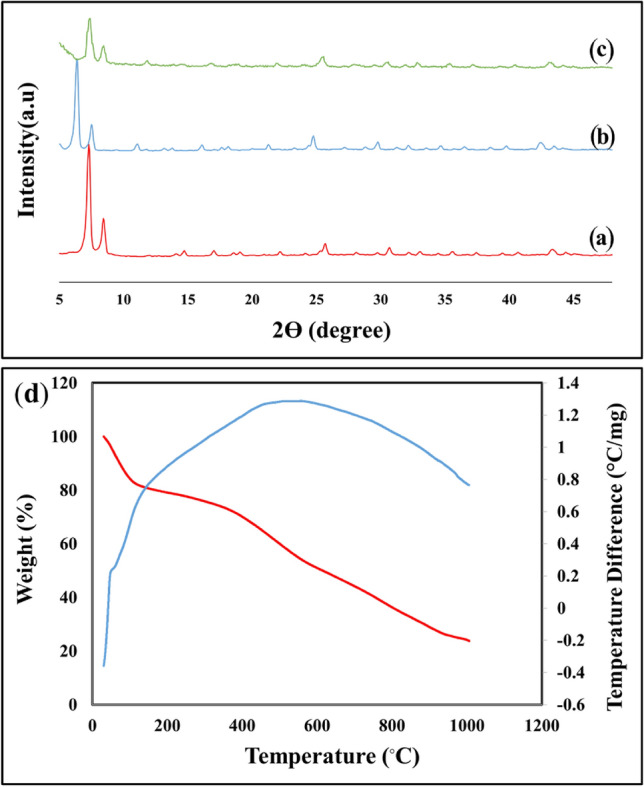
XRD patterns of (a) UiO-66-NH_2_, (b) UiO-66-Pyca, (c) UiO-66-Pyca-Ce (III) and (d) TGA-DTA curves of the UiO-66-Pyca-Ce (III) catalyst.

Two main weight losses were discovered during the thermal analysis of the UiO-66-Pyca-Ce (III) catalyst, which were supported by TGA-DTA curves (Fig. 3d). The first mass loss started when the temperature reached 120 °C. The solvent molecules trapped in the holes and the water molecules absorbed on the surface of the MOF are responsible for this 20% decrease, which results in a broad endothermic peak in this region of the DTA curve. The second 20% drop was noticed in the 120 to 500 °C temperature range. The presence of aliphatic groups, unreacted molecules, the breakdown of organic compounds, and cerium species are thought to be the causes of this reduction. Water molecules that were guests in big cages have also relaxed, which is another reason for the decrease. The effect of this reduction can also be seen in the DTA curve and the strength of its exothermic peak. Decomposition of the framework occurs at a temperature higher than 550 °C, whereas UiO-66-Pyca-Ce (III) exhibits thermal stability up to 500 ℃. Inductively coupled plasma-atomic emission spectrometry (ICP-AES) was also performed for UiO-66-Pyca-Ce (III), which revealed that the resulting compound contained 4.350 mol% Ce and 30.372 mol% Zr.

#### SEM images and elemental mapping analysis

**Figure 4:**
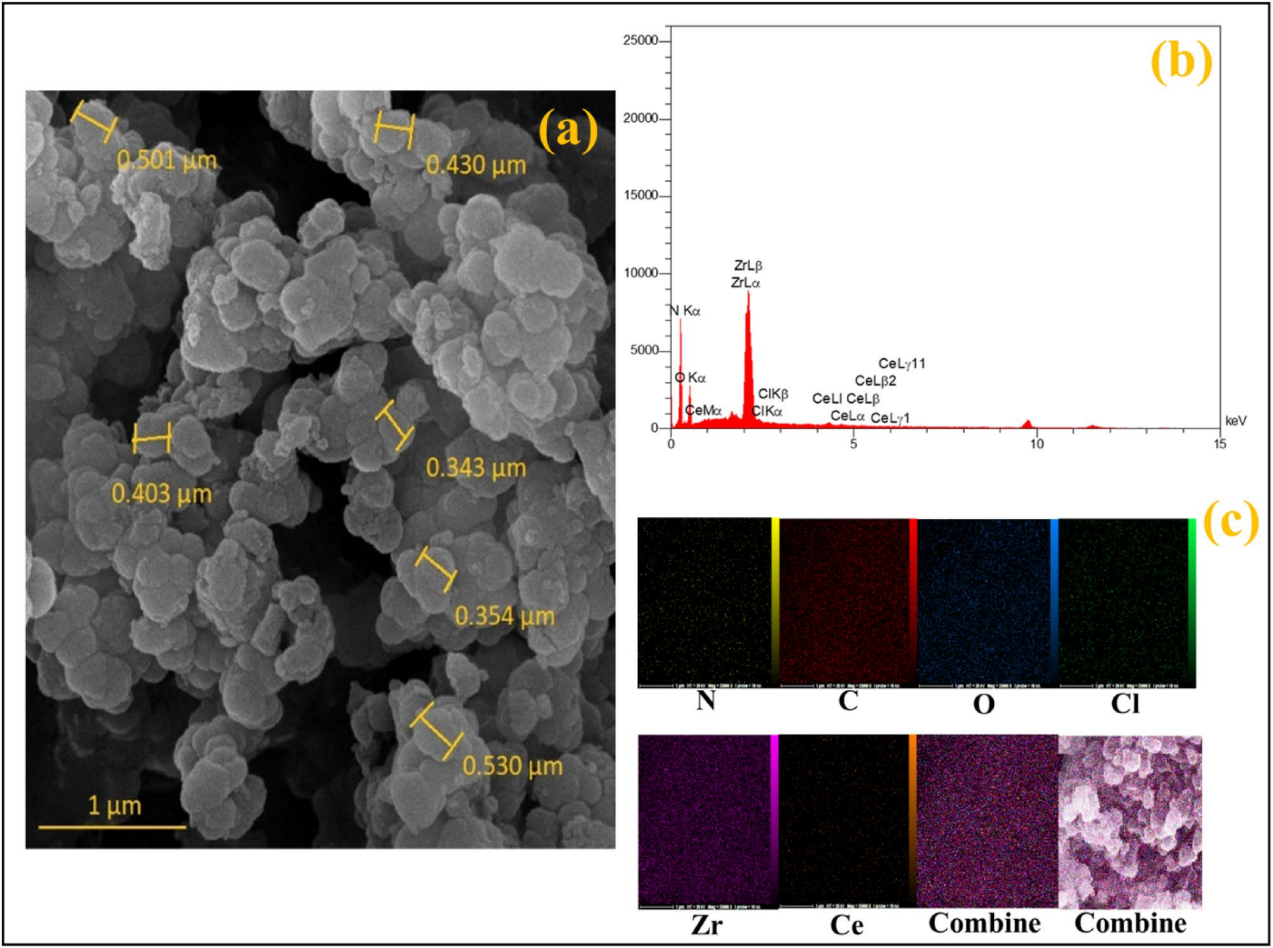
(**a**) SEM images of UiO-66-Pyca-Ce (III, (**b**) EDX analysis of UiO-66-Pyca-Ce (III) and (**c**) The elemental mapping analysis of the UiO-66-Pyca-Ce (III).

SEM images of UiO-66-Pyca-Ce (III) reveal that the catalyst’s structure remains unchanged after the modification during the functionalization process. Figure 4 demonstrates that the crystal size distribution appears uniform (Fig. 4a). Additionally, energy-dispersive X-ray (EDX) analysis confirms the presence of cerium in UiO-66-Pyca-Ce (III) (Fig. 4b). Figure 4c demonstrates the elemental mapping analysis of the UiO-66-Pyca-Ce (III) catalyst. This figure further confirms the continuous dispersion of Ce, Zr, N, O, and C elements on the catalyst.

#### BET analysis

**Figure 5:**
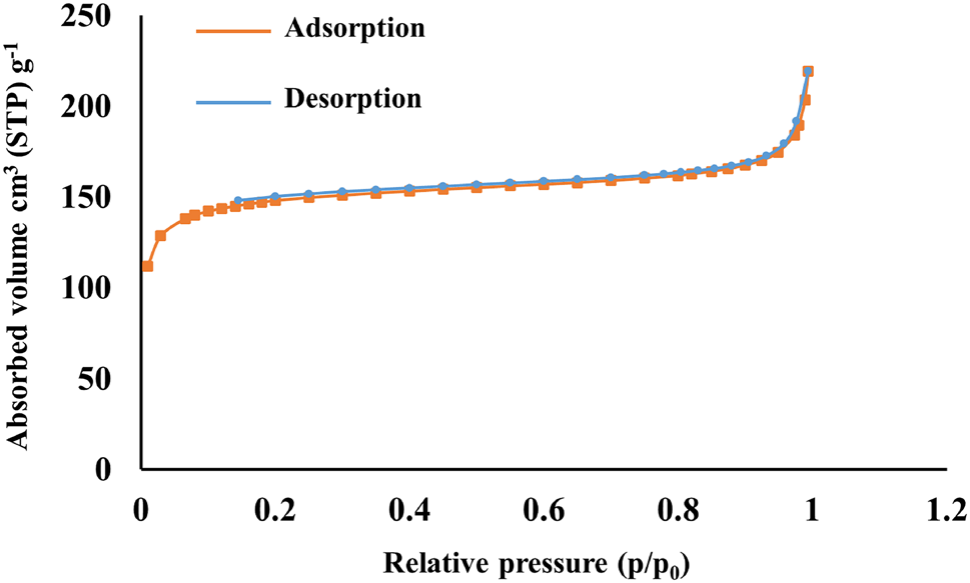
BET analysis of UiO-66-Pyca-Ce (III).

The nitrogen adsorption–desorption isotherm of UiO-66-Pyca-Ce (III) is shown in Fig. 5. As seen in the Figure; each sample exhibits a type I isotherm. The Langmuir surface area of UiO-66-Pyca-Ce (III) was determined to be 501.63 m^2^/g, with a pore volume of 0.28 cm^3^/g and a pore size of 2.27 nm. These results indicate that a relatively high surface area and the presence of pores are necessary for constructing an effective catalyst.

### Catalytic application evaluation of UiO-66-Pyca-Ce (III)

#### Optimization of factors affecting the one-pot synthesis of polyhydroquinolines derivatives

To identify the ideal reaction conditions, a number of variables affecting the one-pot, four-component condensation reaction of polyhydroquinoline derivatives were examined (Table 1, entries 1–10). The model reaction consisted of a mixture of benzaldehyde (1 mmol), ethyl acetoacetate (1 mmol), dimedone (1 mmol), and ammonium acetate (2 mmol), performed at ambient temperature. Initially, without UiO-66-Pyca-Ce (III) present, no significant progress was observed in the reaction (Table 1, entry 1). Subsequently, the effect of different parameters such as solvent type, reaction time, reaction conditions, and the amount of UiO-66-Pyca-Ce (III) was investigated (Table 1, entries 2–10). Under fixed conditions with UiO-66-Pyca-Ce (III), the model reaction was conducted at room temperature and under reflux conditions (Table 1, entries 2–3). It was found that the ambient temperature provided optimal reaction conditions, resulting in shorter reaction times and higher efficiency (Table 1, entry 3). Additionally, using ethanol instead of water as the reaction's solvent produced noticeably better results (Table 1, entry 3). The model reaction was performed with varying amounts of UiO-66-Pyca-Ce (III), and based on the results, the most optimal amount was determined to be 0.01 g (Table 1, entry 3), considering that a lower value is preferable. Therefore, based on these investigations, it was determined that conducting the model reaction at room temperature, using ethanol as the solvent, and using 0.01 g of UiO-66-Pyca-Ce (III) were the best conditions.

**Table 1:** Optimizing various factors based on model reaction^a^.

Entry	Catalyst (g)	Solvent	Condition/temperature (˚C)	Time (h:min)	Yield^b^ (%)
1	–	EtOH	r.t./25	00:15	N.R
2	0.01	EtOH	Reflux/80	00:08	90
3	0.01	EtOH	r.t./25	00:08	90
4	0.01	C_3_H_6_O	r.t./25	00:08	80
5	0.01	H_2_O	r.t./25	00:08	74
6	0.01	CH_2_Cl_2_	r.t./25	00:08	84
7	0.005	EtOH	r.t./25	00:04	84
8	0.015	EtOH	r.t./25	00:10	90
9	0.020	EtOH	r.t./25	00:12	88
10	0.025	EtOH	r.t./25	00:06	85

^a^benzaldehyde (1 mmol), ethyl acetoacetate (1 mmol), ammonium acetate (2 mmol), and dimedone (1 mmol); ^b^Isolated yield.

To generalize the optimal reaction conditions and evaluate the performance and yield of UiO-66-Pyca-Ce (III) as a catalyst, an extensive range of substituted aldehydes (1 mmol) with both electron-donating and electron-withdrawing substituents, along with dimedone (1 mmol), ethyl acetoacetate (1 mmol), and ammonium acetate (2 mmol), were used (Table 1, entries a-f). Diverse substituted polyhydroquinoline derivatives (5a-f) were synthesized using 0.01 g of UiO-66-Pyca-Ce (III) at room temperature. The outcomes shown in Table 2 resulted that the desired products were synthesized in a brief amount of time with a high yield. Based on the relevant results, it can be deduced that the UiO-66-Pyca-Ce (III), at room temperature, resulted a successful synthesis of products with a high yield in a short amount of time.

**Table 2:** Synthesis of polyhydroquinoline derivatives using UiO-66-Pyca-Ce (III) as a catalyst^a^.


Entry	Product	R1	Time (min)	Yield^b^ (%)	Melting point (℃)	
					Observe	Ref
5a			7	92	235–238	^55^
5b			7	94	172–174	^56^
5c			10	90	223–225	^55^
5d			12	89	254–256	^56^
5e			8	90	199–200	^57^
5f.			6	96	249–252	^56^

^a^Reaction conditions: (1) Substituted aldehyde (1 mmol), (2) ethyl acetoacetate (1 mmol), (3) dimedone (1 mmol), (4) ammonium acetate (2 mmol), UiO-66-Pyca-Ce (III) (0.01 g), ambient temperature; ^b^Isolated yield.

#### Assessment of UiO-66-Pyca-Ce (III) in the synthesis of polyhydroquinoline derivatives in comparison with other introduced researches

In Tables 1 and 2, the obtained results are presented to evaluate the efficiency and performance of UiO-66-Pyca-Ce (III) in the synthesis of polyhydroquinoline derivatives by replacing a wide range of aldehydes (electron-withdrawing or electron-donating). In order to evaluate the effectiveness and performance of UiO-66- Pyca-Ce (III), Table 3 compares UiO-66- Pyca-Ce (III) with other prior catalysts for the synthesis of polyhydroquinoline derivatives. For this, a number of variables have been taken into account, such as the quantity of catalyst, type of solvent, temperature during the reaction, reaction time, and the proportion of isolated products. The results are summarized in Table 3, entries 1–5. In addition to the advantages, the reported data have certain disadvantages, including the high cost of the catalyst used, toxic and volatile solvents, harsh reaction conditions such as long reaction times and high temperatures, and low product yields (Table 3, entries 1–4). However, compared to prior studies, UiO-66-Pyca-Ce (III) demonstrates unique and distinct advantages. Its proficiency allows for synthesizing polyhydroquinoline derivatives with a lower catalyst dosage, shorter reaction times, and high efficiency (Table 3, entry 5).

**Table 3:** Comparison of UiO-66-Pyca-Ce (III) catalyst with other reported investigations^a^.

Entry	Catalyst	Amount of catalyst (g)	Solvent	Temperature (°C)	Time (h:min)	Yield^b^ (%)	Ref
1	CTAB	10 mol%	H2O	Reflux	1:30	85	^10^
2	Palladium NPs	0.04 mmol	THF	Reflux	4:00	89	^58^
3	GSA@Fe3O4 MNPs	0.05	EtOH	Reflux/80	4:00	90	^59^
4	L*-*proline	10 mol%	EtOH	Reflux	6:00	92	^13^
5	UiO-66-Pyca-Ce (III)	0.01	EtOH	r.t./25	00:06	90	This work

^a^Reaction mixture: benzaldehyde (1 mmol), ethyl acetoacetate (1 mmol), dimedone (1 mmol), ammonium acetate (2 mmol); ^b^Isolated yield.

#### Proposed mechanism for synthesis of polyhydroquinoline derivatives

**Figure 6:**
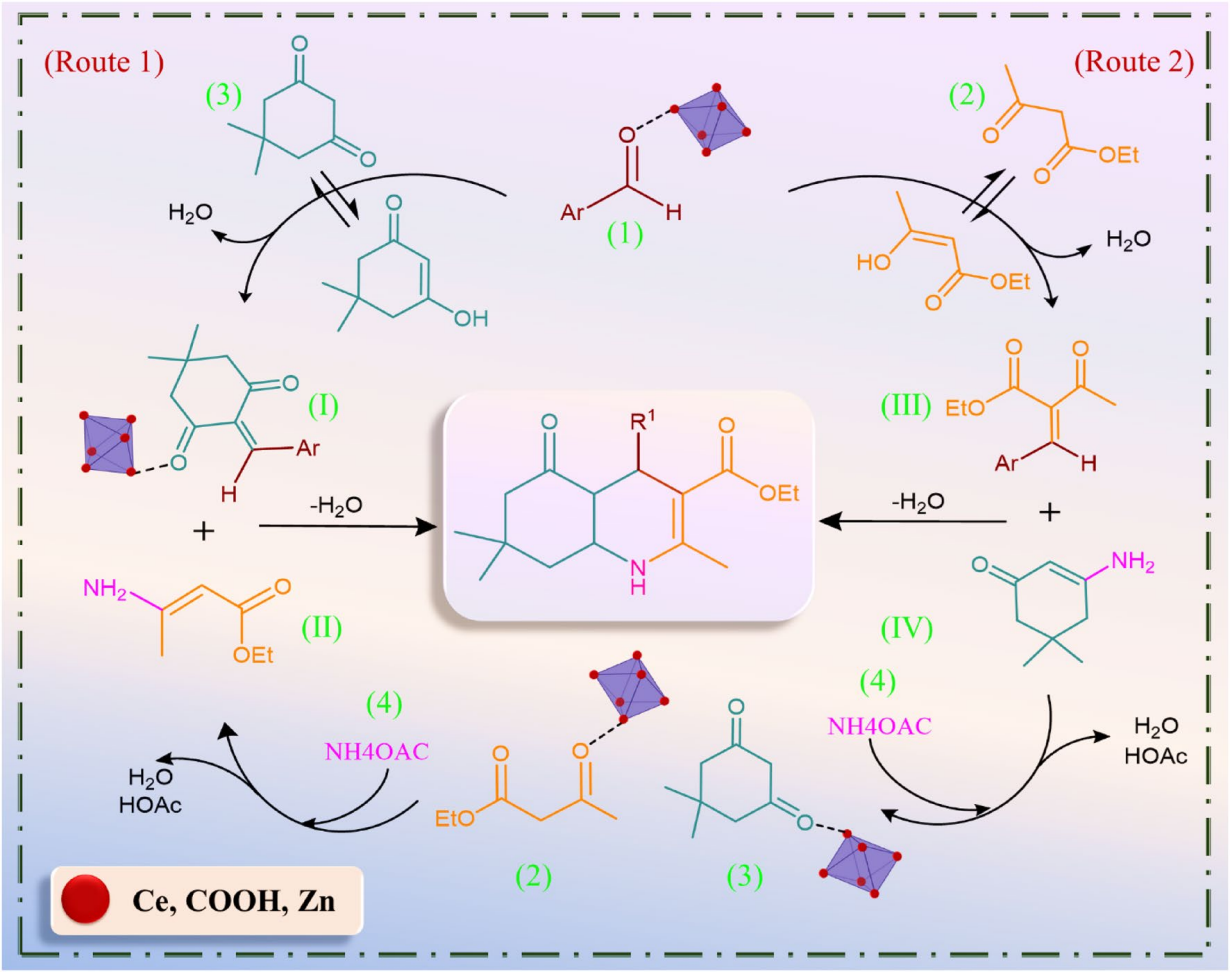
Proposed mechanism for the synthesis of polyhydroquinoline derivatives in the presence of the UiO-66-Pyca-Ce (III).

This study identified UiO-66-Pyca-Ce (III) as the primary catalytic agent driving the synthesis reaction of polyhydroquinoline derivatives forward. The proposed mechanism shown in Fig. 6 can be explained in two different ways, according to earlier studies. In the first method, intramolecular interactions between the substituted aldehyde and the carbonyl group of ethyl acetoacetate activate each other. This leads to a Knoevenagel condensation reaction between the activated substituted aldehyde (**1**) and the enol dimedone (**3**), resulting in the formation of intermediate (**I**). Simultaneously, intermediate (**II**) is formed from the reaction between activated ethyl acetoacetate (**2**) and ammonium acetate (**4**). Finally, a Michael addition reaction occurs between intermediates (**I**) and (**II**), followed by cyclization and elimination of water, leading to the desired polyhydroquinoline derivatives (**5a-f**). In the second method, intermediate (**III**) is initially created in the Knoevenagel reaction between the active aldehyde (**1**) and the enol form of ethyl acetoacetate (**2**). Intermediate (**IV**) is also produced by the reaction between activated dimedone (**3**) and ammonium acetate. Similarly, to the first method, a Michael addition reaction occurs between intermediates (**III**) and (**IV**), followed by cyclization and elimination of water, resulting in the desired polyhydroquinoline derivatives (**5a-f**). For further explanation, it can be said that the synthesized catalyst is an acidic catalyst, which generally activates electrophiles. In the case of aldehydes, either cerium or zirconium with vacant orbitals will perform this activation. They coordinate with the oxygen of the aldehyde, weakening the carbonyl double bond. Alternatively, the oxygen of the aldehyde forms a hydrogen bond with COOH groups, leading to the weakening of the carbonyl double bond. The weakened carbonyl then becomes vulnerable to attack. These events happen for ethyl acetoacetate and dimedone in both the first and second routes, and are followed by a tautomerization procedure that adds a double bond. The double bond has the potential to engage in nucleophilic attack on the activated aldehyde. In the subsequent steps, the dimedone and/or ethyl acetoacetate undergo similar activation processes, and ammonium acetate attacks the activated species.

#### Reusability assessment of UiO-66-Pyca-Ce (III) after several catalytic performances

**Figure 7:**
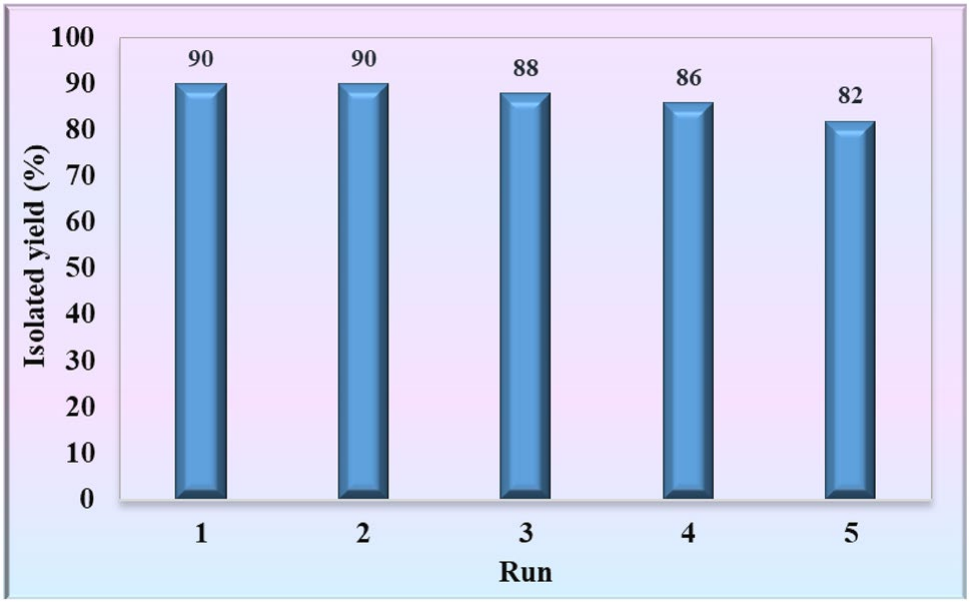
Reusability study of UiO-66-Pyca-Ce (III).

Recyclability and reuse of catalysts, whether in industrial applications or the discussion of green chemistry, are essential and vital for developing of chemical reactions. Therefore, in this study, the reusability of UiO-66-Pyca-Ce (III) in the synthesis of polyhydroquinoline derivatives was investigated. After each reaction, the catalyst was detached through washing and centrifugation, and then dried. The results are depicted in Fig. 7, and they show that the catalyst can used as a qualified catalyst even after being used five times without significantly reducing the efficiency of the desired products.

## Conclusions

In this study, a catalyst was fabricated using a solvothermal reaction based on zirconium MOF that had been modified with pyridine carboxaldehyde and cerium metal. UiO-66-Pyca-Ce (III) was created and introduced as an effective catalyst with noteworthy features for the synthesis of heterocyclic compounds. The structural properties of this catalyst were assessed through various analyses, including XRD, TGA, FE-SEM, ICP, and BET. Verification of the presence of functional groups, analysis of its main constituent elements, and their dispersion were performed through FT-IR, Far IR, and EDX analysis, respectively. This catalyst demonstrated high efficiency in producing the desired product in the shortest amount of time at room temperature, without requiring special conditions, for a number of reasons, including three Lewis acid activating functions. These features show promising potential for the manifestation of a highly efficient catalyst, with a yield of 90%, particularly in the asymmetric synthesis of polyhydroquinoline derivatives. In addition to the benefits above, considering the importance of catalyst recovery, reusability in the industry, and its impact on the environment, the efficiency of the produced catalyst was evaluated multiple times. The results demonstrated the catalyst's pleasant stability and performance in reuse.

## Data Availability

The datasets generated and/or analyzed during the current study are available at the http://www.crystallography.net/cod/4348132.cif.
